# Width dependence of the 0.5 × (2e^2^/h) conductance plateau in InAs quantum point contacts in presence of lateral spin-orbit coupling

**DOI:** 10.1038/s41598-019-48380-1

**Published:** 2019-08-21

**Authors:** Partha Pratim Das, Marc Cahay, Shashikala Kalita, Sib Sankar Mal, Alok Kumar Jha

**Affiliations:** 1 Department of Physics, National Institute of Technology Karnataka, Surathkal, 575025 India; 2 Spintronics and Vacuum Nanoelectronics Laboratory, University of Cincinnati, Cincinnati, Ohio 45040 USA; 3 Department of Electronics and Communication Engineering, Tezpur University, Tezpur, Assam 784028 India; 4 Department of Chemistry, National Institute of Technology Karnataka, Surathkal, 575025 India; 5 Department of Materials Science and Engineering, Kyushu Institute of Technology, Tobata-ku, Kitakyushu 804-8550 Japan

**Keywords:** Engineering, Nanoscience and technology

## Abstract

The evolution of the 0.5G_o_ (G_o_ = 2e^2^/h) conductance plateau and the accompanying hysteresis loop in a series of asymmetrically biased InAs based quantum point contacts (QPCs) in the presence of lateral spin-orbit coupling (LSOC) is studied using a number of QPCs with varying lithographic channel width but fixed channel length. It is found that the size of the hysteresis loops is larger for QPCs of smaller aspect ratio (QPC channel width/length) and gradually disappears as their aspect ratio increases. The physical mechanisms responsible for a decrease in size of the hysteresis loops for QPCs with increasing aspect ratio are: (1) multimode transport in QPCs with larger channel width leading to spin-flip scattering events due to both remote impurities in the doping layer of the heterostructure and surface roughness and impurity (dangling bond) scattering on the sidewalls of the narrow portion of the QPC, and (2) an increase in carrier density resulting in a screening of the electron-electron interactions in the QPC channel. Both effects lead to a progressive disappearance of the net spin polarization in the QPC channel and an accompanying reduction in the size of the hysteresis loops as the lithographic width of the QPC channel increases.

## Introduction

Exploring viable route and device architecture to create, manipulate and detect spin-polarized current through *all-electric means* has received considerable attention over the last decade^1–3^. Spin-orbit coupling (SOC) offers numerous interesting ways in this direction in variety of device architectures made from two-dimensional electron gas (2DEG) systems^4^. Among others quantum point contacts (QPCs) have shown promising applications for spin based devices^5–41^.

For more than two decades, conductance anomalies have been observed experimentally in QPCs at non-integer multiples of *G*_0_ (=*2e*^2^*/h*)^42–45^. Thomas *et al*.^42^ suggested that the 0.7*G*_0_ conductance anomaly may be due to a spontaneous ferromagnetic spin polarization in the QPC constriction. Theoretical explanations for the conductance anomalies include the onset of spontaneous spin polarization in the narrow portion of the QPC as a result of the exchange-correlation interaction^46–48^, the presence of quasi-bound states^49^ being formed in the QPC constriction, and the Kondo effect^50^, among others^43–45^.

Crook *et al*.^41^ observed a 0.5G_o_ conductance plateau in a symmetric GaAs QPC with symmetrically biased top split-gates, which they also attributed to the onset of spontaneous spin-polarization. The plateau was found to be more pronounced when the QPC potential energy landscape was made *symmetric* with the proximity of a low-temperature scanning probe. Over the last few years, spin polarization in a QPC channel and the accompanying 0.5G_o_ conductance plateau have been reported by several groups when an *asymmetric bias* is applied between the top or in-plane side-gates (SGs) of a QPC. The 0.5G_o_ conductance plateau has been found in QPCs made of different materials (GaAs and InAs) with 2DEG of different electron mobility, and with a wide variety of heterostructure design (including suspended QPCs) and QPC dimensions^20–41^.

Specifically, over the last decade, we have experimentally observed (in both InAs and GaAs 2DEG based QPCs) conductance plateaus around 0.5G_0_ by tuning the electrostatic confining potential of the QPC constriction using an asymmetric bias on its side-gates (SGs)^20,27–31^. We argued that onset of such a conductance anomaly constitutes indirect evidence of the creation of spontaneous spin-polarized current in the narrow portion of the QPCs. It was shown that lateral spin-orbit coupling (LSOC), due to the lateral in-plane electric field of the confining potential of the QPCs with in-plane SGs, is an efficient way to generate a strongly spin-polarized current by *purely electrical means*^20,27–31^. Furthermore, we used Non-Equilibrium Green’s Function (NEGF) analysis to model both InAs and GaAs QPCs with SGs in the presence of LSOC. Assuming a parabolic dispersion relation for the conduction band in the InAs QPC channel, the following three conditions were found to be *sufficient* to create a strong spin polarization^20,51^: (1) an asymmetric lateral confinement in the narrow portion of the QPC, (2) a LSOC due to the lateral confining potential of the QPC and (3) a strong electron-electron (e-e) interaction. More importantly, NEGF simulations suggest that a very strong SOC is not paramount for achieving a strong spin polarization. In fact, we have observed 0.5G_0_ conductance plateaus in both GaAs and InAs QPCs with very different LSOC strength^20,27,28^.

The effects of LSOC in a one-dimensional asymmetrically biased quantum wire were studied by Karlsson *et al*.^52^ using a Hartree–Fock method. They showed that spin polarization can be generated by LSOC in the presence of a non-zero source-to-drain bias. The latter was shown to be due to either numerical noise mimicking the effects of a random magnetic field generated by the metallic contacts to the device or due to a random background magnetic field such as the earth magnetic field. Karlsson *et al*. showed that electrons spontaneously form spin texture in the quantum wire with an associated finite spin polarization as a result of e-e interaction. They also found that the spin polarization direction is random at zero source-drain bias and that LSOC influences the spin rows orientation only when a non-zero source-to-drain bias is applied across the quantum wire.

Recently, Chuang *et al*. achieved near 100% spin injection and detection in an all-electric AlGaAs/GaAs spin valve made of asymmetrically biased QPCs with top gates^33^. Chuang *et al*. argued that the asymmetry in LSOC in their devices is ultimately responsible for the efficient spin injection and detection. Very recently, Zhdanov *et al*.^39^ and Pokabov *et al*.^40^ performed conductance measurements on unsuspended and suspended GaAs QPCs with in-plane SGs and the 0.5G_o_ conductance plateau was only observed for the latter. They argued that in their suspended QPCs spin transport mechanism was also governed by LSOC and that the onset of 0.5G_o_ plateau in asymmetrically biased QPCs is due to enhanced e-e interaction, when the QPCs were suspended. These observations further support the argument that it is the strong e-e interaction which is instrumental for the appearance of 0.5G_o_ plateau. These reports further suggest that the onset of half-integer conductance plateau can be treated as indirect evidence of spin-polarization in QPC constrictions.

To date, only a few groups have reported theoretical and experimental investigations of the presence of hysteresis in electrical characteristics of QPCs^9,53–61^. Shailos *et al*. studied conventional QPC devices with an additional gate whose purpose was to modify the electron density on one side of the device and break the QPC symmetry in a controlled manner^54^. As the bias on this additional finger gate was varied, reproducible conductance anomalies below the normal conductance plateau G_o_ were found. In addition, alterations to the normal conductance plateaus were also observed. Furthermore, hysteresis was observed in the conductance characteristics while varying the split-gate voltage in opposite directions. Starting with a density functional theory, Ihnatenska and Zozoulenko calculated the conductance of a typical QPC in the presence of a symmetric potential applied to its split-gates^9^. The latter is responsible for lifting the spin degeneracy of the conductance channels and a broad conductance plateau near 0.5G_0_. Their calculations also predict the onset of hysteresis in the conductance curves when forward and reverse sweeps are applied to the QPC split-gates – a feature attributed to the presence of quasi bound states in the narrow portion of the QPC. Finally, hysteresis in the conductance characteristics of QPCs can be generated by surface acoustic wave, as recently shown by Song *et al*.^56^. The surface acoustic wave is responsible for a modification of surface state distribution on the QPC sidewalls which is ultimately responsible for the observed hysteresis^56^.

In the past, we observed hysteresis when sweeping the common mode bias, V_G_, applied to the two in-plane SGs of asymmetrically biased GaAs QPC^57,58^ in both directions. The hysteresis loops were found to be larger when the amount of bias asymmetry Δ*V*_G_ between the SGs was increased and to be dependent on the Δ*V*_G_ polarity. These results are in agreement with NEGF simulations which show that the conductance plots versus common gate voltage applied to a QPC SGs reveal the presence of single or multiple hysteresis loops when varying the QPC dimensions and biasing conditions^57,58^. The following features were predicted by NEGF simulations: (a) hysteresis in the conductance plots is present only for sufficiently long QPCs and the e-e interaction in the channel must be strong enough; (b) The size of the hysteresis loops depends both on the polarity and magnitude of the asymmetric bias between the side gates, Δ*V*_G_, and also on the magnitude of the e-e interaction; (c) The hysteresis loops are sensitive to the presence of dangling bonds on the QPC sidewalls^59^. We also used NEGF simulations to illustrate how to fine tune the location of the 0.5 G_0_ and the onset of hysteresis loops in four-gate QPCs when LSOC is present in the channel^60^. The rich plethora and sensitivity of the hysteresis loops is a fingerprint of the wide variety of metastable spin textures accompanying the onset of a net spin polarization and the appearance of conductance anomalies in the QPC constriction^58,59^. The observation of hysteresis loops is yet another indirect evidence for the onset of spontaneous spin polarization in the QPC constriction. Previously, we have shown that the observed hysteresis loops depend on the sweep rate of the common mode potential applied to the QPC SGs^58,59^.

In a recent study^30^ using InAs QPCs with identical lithographic width but longer channel length, we found that there is approximately a fourfold increase in the range of common sweep voltage over which the 0.5G_0_ conductance plateau is observed when the QPC aspect ratio (ratio of lithographic length over lithographic width of the narrow portion of the structure) increases by a factor of 3. NEGF simulations indicate that the observation of 0.5G_0_ conductance plateau over a larger range of common sweep voltage is due to an increased importance of the effects of e-e interactions in QPC devices with larger aspect ratio (longer length for fixed width)^30^.

## Methods

**Figure 1:**
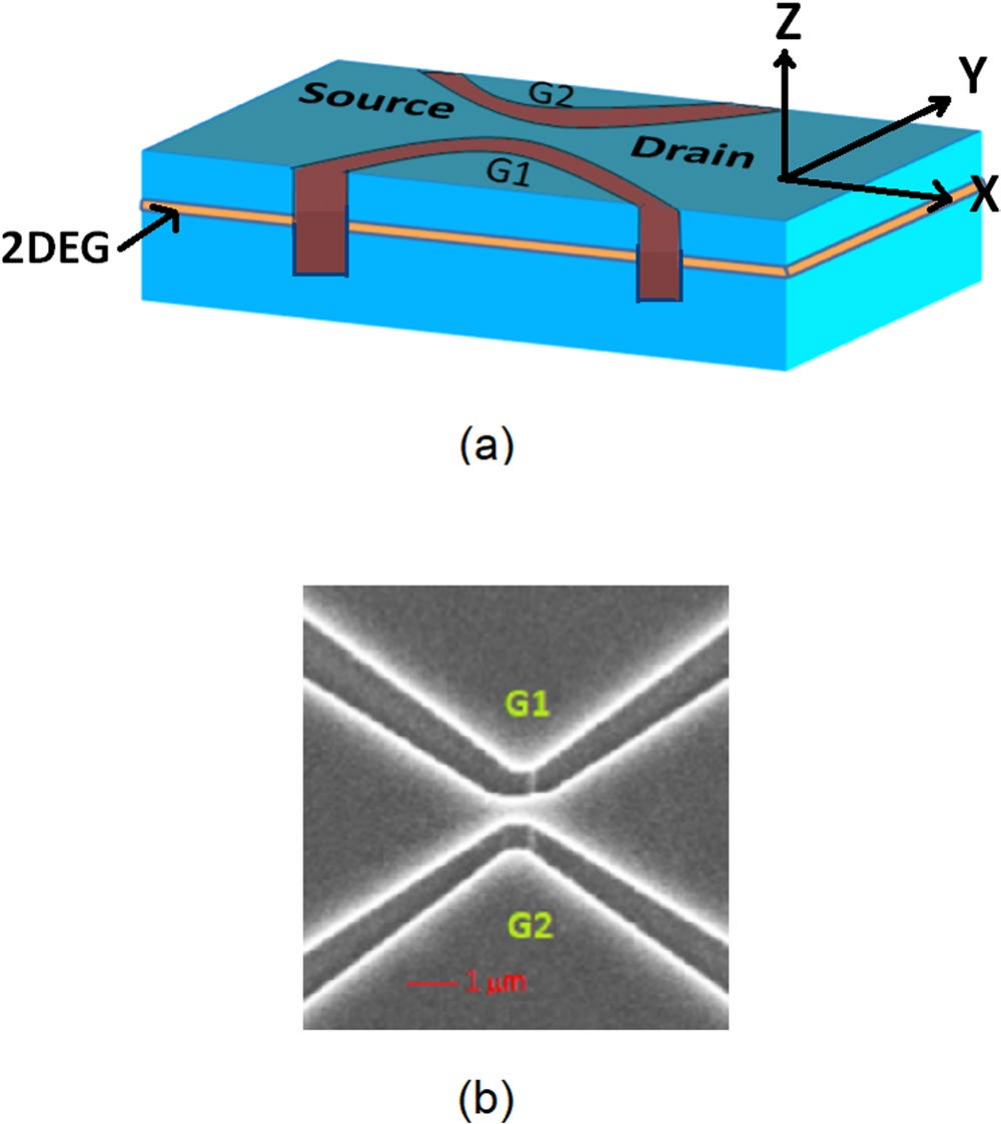
(**a**) Schematic representation and (**b**) scanning electron micrograph (SEM) of an InAs based QPC with two in-plane SGs (G1 and G2). Dark areas are the deep-etched isolation trenches defining the two SGs. The current flows in the x-direction. Application of an asymmetric bias potential between the SGs leads to a lateral electric field along y-direction. The latter is responsible for LSOC in the QPC narrow portion. The 2DEG lies on the xy-plane. The z axis is the direction of growth of the heterostructure.

In this work, we perform a systematic study on how the size of the hysteresis loops observed in the conductance measurements during forward and reverse sweeps evolves in a number of asymmetrically biased side-gated InAs QPCs (with different lithographic channel widths while keeping their lithographic channel length fixed). QPC devices were fabricated using a high-electron-mobility InAs/InAlAs quantum well based 2DEG. The details of the quantum well structure and QPC fabrication process are described elsewhere^30^. Shubnikov-de Haas and quantum Hall measurements at 4.2 K were used to determine the carrier concentration and electron mobility of the 2DEG which were found to be equal to 2.2 × 10^16^/m^2^ and 3.67 m^2^/Vs, respectively^30^. As shown in Fig. 1, electron beam lithography and a wet etching technique were used to define deep trenches in the 2DEG to form narrow QPC constrictions^30^.

We fabricated seven QPCs with different aspect ratios (i.e., ratio of lithographic width over lithographic length). Figure 1a,b represent a schematic and a scanning electron micrograph of one of our fabricated QPC structures with in-plane SGs (G1 and G2), respectively. In QPC 1 through 7, the narrow portion had the same lithographic length (L ~ 890 nm) but different lithographic widths (W) varying from 220 nm, 260 nm, 330 nm, 410 nm, 520 nm, 640 nm, and 780 nm, respectively. This corresponds to a QPC aspect ratio (W/L) ranging from 0.247, 0.292, 0.371, 0.461, 0.584, 0.719 to 0.876 for QPC 1 through 7, respectively. The reason the maximum lithographic length of the channel for all QPC devices was maintained around 890 nm is because InAs has a measured electron spin coherence length less than a micron at 4.2 K^61^. In fact, one of our earlier reports^30^ has shown the existence of the broadest 0.5G_o_ plateau for an InAs QPC having lithographic channel length around 930 nm. We varied the electrostatic width of the QPC channel by applying a bias to the ohmic in-plane SGs. This leads to a depletion of the channel in the narrow portion of the QPC. DC bias was applied to maintain fixed negative voltages *V*_G1_ and *V*_G2_ on the two SGs, as shown in Fig. 1b. The QPC devices were first cooled down to liquid nitrogen (77 K) and then to liquid helium (4.2 K). Further details of our conductance measurements are provided elsewhere^30^. The QPC conductance was measured as a function of a common sweep voltage, *V*_G_, applied to the two SGs. A four-probe lock-in technique with a drive frequency of 17 Hz was used to measure the linear conductance *G* (=*I*/*V*_*ds*_) of the QPC channel for different bias asymmetry Δ*V*_G_ while varying the common sweep voltage *V*_G_ applied to the two SGs. In all conductance measurements performed at 4.2 K, a small source-to-drain voltage of *V*_*ds*_ = 100 μV was applied across the QPC. For all QPCs, the negative voltages *V*_G1_ and *V*_G2_ applied to the two SGs were varied until a robust 0.5G_o_ plateau was observed as the common gate voltage *V*_G_ was continuously swept^29^.

## Results

**Figure 2:**
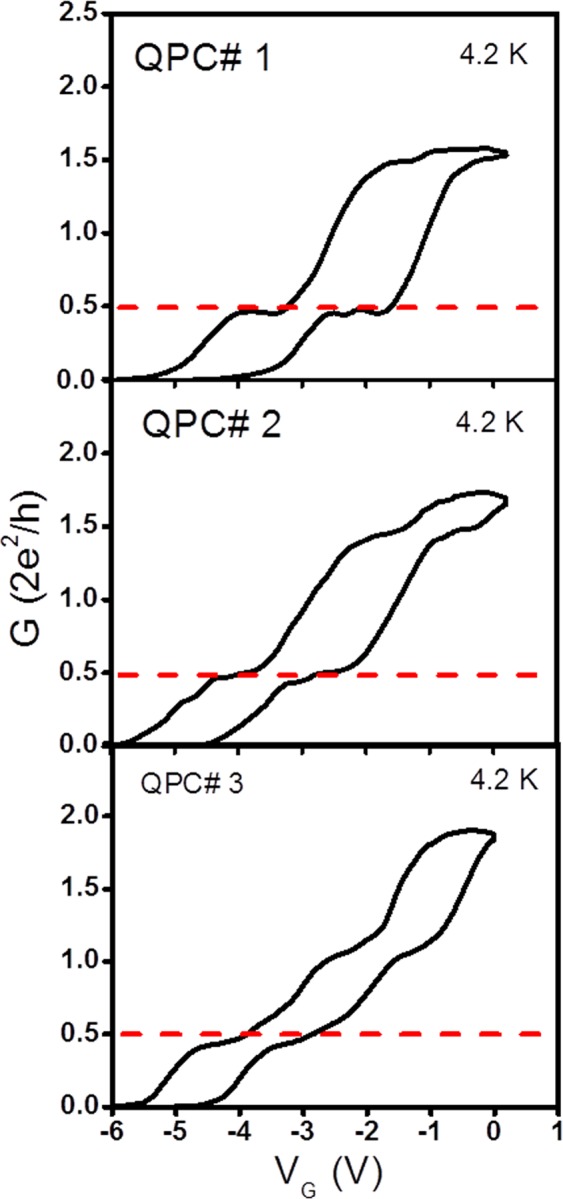
Forward (blue arrows) and reverse sweeps (green arrows) in conductance G (in units of *2e*^2^*/h*) plots for QPCs 1 through 3. Although the individual pinch-off voltage for each QPC channel is different from one another, the range of common gate signal (V_G_) is kept the same for all devices for clarity. All conductance measurements were taken at T = 4.2 K. V_G_ is the common mode voltage superimposed on initial side gate potentials V_G1_ and V_G2_ applied to generate the asymmetric potential energy profile in the narrow portion of the QPC.

**Figure 3:**
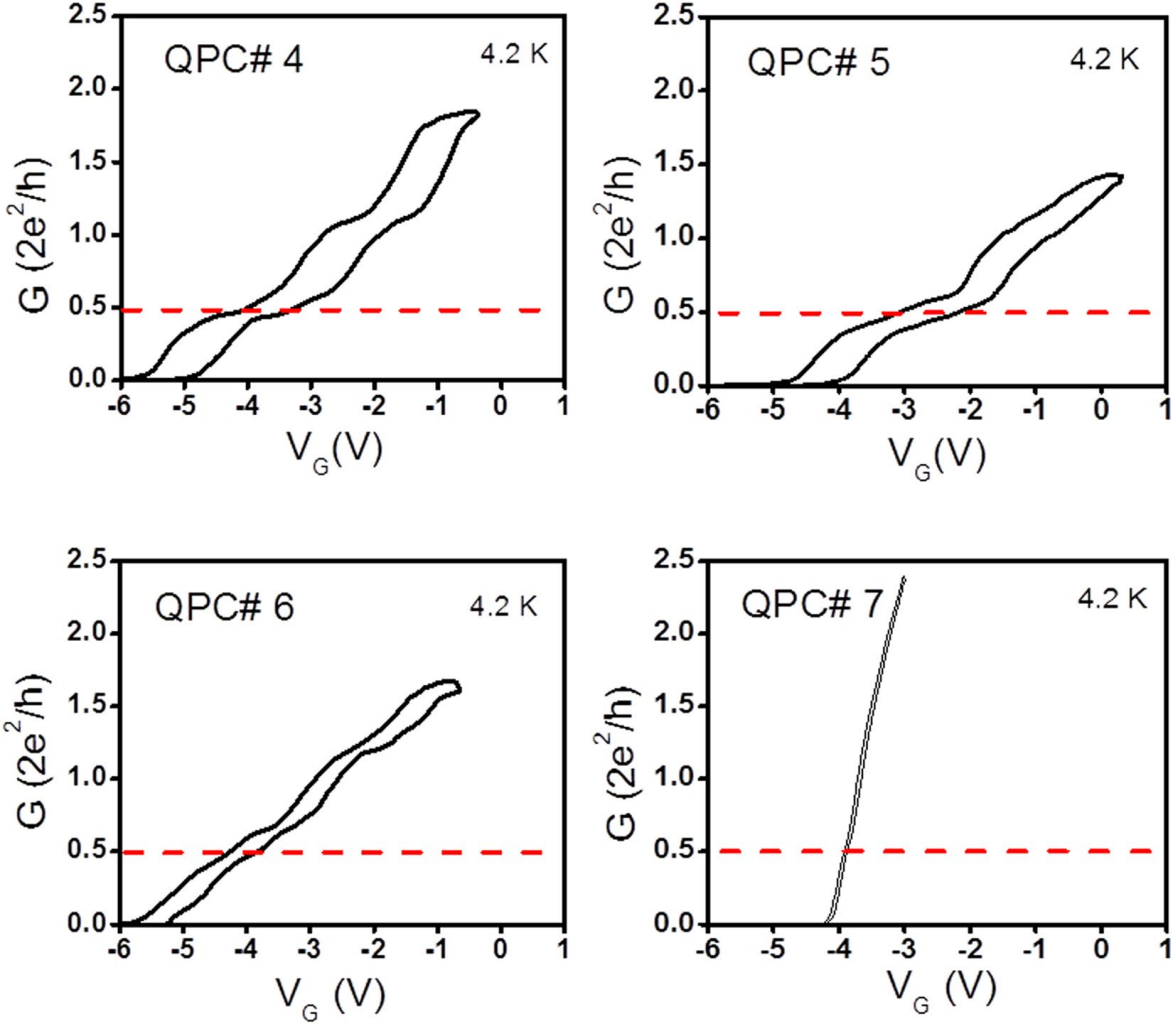
Forward (blue arrows) and reverse sweeps (green arrows) in conductance G (in units of *2e*^2^*/h*) plots for QPCs 4 through 7, as in Fig. 2.

Figures 2 and 3 show the common gate voltage (V_G_) dependence of the conductance for the forward (blue arrows) and reverse sweeps (green arrows) for QPCs 1 through 3 and QPCs 4 through 7, respectively. For ease of comparison of the conductance plots, the range of V_G_ was kept same for all QPCs. Figures 2 and 3 show that the threshold voltage in the forward sweep, i.e., the value V_G_ at which the conductance plot rises depends on the QPC dimensions.

Focusing on the conductance behavior near the 0.5G_0_ conductance plateau, it can be seen that the latter is more pronounced for QPC1 with the smallest channel width and gradually disappears as the QPC width increased. For QPC7, the conductance plot is close to linear and there is no remnant of the original conductance plateau as observed for QPC1. The conductance plot for QPC7 is indicative of a near ohmic behavior for the QPC with the largest width. Figures 2 and 3 show that the hysteresis loop size in the conductance plots also diminishes as the QPC width increases. QPCs 1 and 2 also have an anomalous conductance plateau around 1.5G_0_ and QPCs 3 and 4 have another conductance plateau slightly above the normal conductance plateau G_0_, typically observed in symmetrically biased QPCs with top split-gates^45^.

Some of the features described above can be explained based on our earlier systematic study of the number and locations of anomalous conductance plateaus in InAs/In_0.52_Al_0.48_As QPCs as a function of the polarity of the common gate voltage between its SGs^29^. Some anomalous conductance plateaus were shown to occur over a range of the common sweep voltage as large as 1 V. A similar trend is observed in the size of the 0.5 G_0_ plateau for QPCs 1 and 2 reported here.

The sensitivity of the conductance measurements to the constant negative voltages *V*_G1_ and *V*_G2_ applied to the two SGs prior to the application of the common sweep voltage V_G_ stems from the importance of surface scattering present on the rugged QPC side walls^29^. Since the 7 QPCs are fabricated with different channel widths, the amount of surface scattering on their side walls will be different leading to both a difference in the threshold voltage in the forward sweep conductance plots and the need for an adjustment in the voltages *V*_G1_ and *V*_G2_ to observe an anomalous conductance plateau around 0.5 G_0_, is listed in column 4 of Table 1.

**Table 1:** Details of the QPC geometrical dimensions (lithographic width and length), aspect ratios (W/L) and the value of the asymmetric bias potential applied on the SGs to achieve the most pronounced anomalous plateaus at conductance value 0.5G_o_ during the forward sweep of the common gate voltage *V*_*G*_.

QPC#	W, L (nm)	Aspect Ratio W/L	(*V*_G1_,*V*_G2_)
QPC 1	220,890	0.247	−2 V, −0.3 V
QPC 2	260,890	0.292	−1 V, −3 V
QPC 3	330,890	0.371	−2.5 V, −5 V
QPC 4	410,890	0.461	−3.2 V, −6.4 V
QPC 5	520,890	0.584	−4.5 V, −7.9 V
QPC 6	640,890	0.719	−3.5 V, −7.8 V
QPC 7	780,890	0.876	−5 V, −9.8 V

The last column gives the applied potential **(***V*_G1_, *V*_G2_) applied to the two SGs before applying the common gate signal *V*_*G*_.

## Discussion

Next, we investigate the physical mechanisms responsible for the progressive disappearance of the 0.5 G_0_ plateau and decrease in size of the hysteresis loops as the width of the QPC channel increases. Since the QPC is fabricated using a nominally symmetric InAs quantum well surrounded by two similar InAlAs barriers of equal height (See Fig. 2 of ref.^30^), the spatial inversion asymmetry is negligible along the heterostructure growth axis and the associated Rashba spin-orbit coupling can be neglected in our QPC structures. Furthermore, the Dresselhaus spin-orbit interaction due to the bulk inversion asymmetry in the direction of current flow is also neglected for simplicity. The major spin-orbit interaction in our QPCs is LSOC due to a lateral confinement provided by the wet etching of the channel to form the QPC and applied potential from the contact gates (See Fig. 1). In the QPC narrow portion, the single-particle Hamiltonian is given by

1 $$\begin{array}{rcl}H & = & {H}_{0}+{H}_{SO}\\ {H}_{SO} & = & \beta \overrightarrow{\sigma }\cdot (\overrightarrow{k}\times \overrightarrow{\nabla }U)=\overrightarrow{\sigma }\cdot {\overrightarrow{B}}_{SO}\end{array},$$

where ${H}_{0}=\frac{1}{2{m}^{\ast }}({p}_{x}^{2}+{p}_{y}^{2})+U(x,y)$, $\beta$ is the intrinsic SOC parameter, $\overrightarrow{\sigma }$ is the vector of Pauli spin matrices and ${\overrightarrow{B}}_{SO}$ is the effective magnetic field induced by the LSOC.

We assume that the 2DEG is located in the (*x*, *y*) plane, where *x* is the direction of current flow from source contact to drain contact and *y* the direction of lateral confinement in the QPC channel; *U*(x, y) is the confinement potential due to both the electrostatic potential on the SGs and the conduction band discontinuity $\Delta {E}_{c}(y)$ at the InAs/vacuum interface. The details of the conductance band energy profile in the vicinity of the QPC constriction, the strength of the LSOC, and the strength of the e-e interaction were analyzed in refs^51,58^.

**Figure 4:**
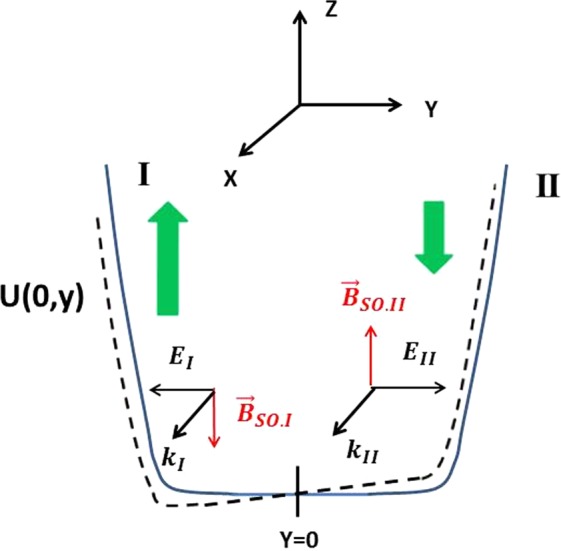
Illustration of the onset of spin polarization in the narrow portion of a QPC in the presence of LSOC when a finite bias is applied between the two SGs. The full and dashed lines represent the potential energy profile U(0,y) in the middle portion of the QPC in the presence of a symmetric and asymmetric bias between the two SGs, respectively. The thick green arrows indicate the amount of spin polarization on either sides of the QPC. Also show are the y-component of the electrical field (${E}_{I}\,$and ${E}_{2}$) near the QPC sidewalls and the associated effective magnetic field ${\overrightarrow{B}}_{SO,I}$ and ${\overrightarrow{B}}_{SO,II}$ on both sides of the QPC channel.

A schematic representation of the confining potential U(0,y) along the y direction in the QPC constriction is illustrated in Fig. 4, where a symmetric (full line) or asymmetric bias (dashed line) is applied on its SGs. The application of the same potential to the two SGs results in effective magnetic field ${\overrightarrow{B}}_{SO}$ which has the same magnitude but opposite directions along the two sidewalls that define the narrowest part of the QPC constriction. Electrons flowing through the QPC constriction with opposite spins experience opposite LSOC forces leading to an accumulation of spins with opposite polarity on the QPC sidewalls (thick green arrows in Fig. 4). The spin-up (spin-down) electrons are the majority spin species on edge I (edge II) and the minority species on edge II (edge I) of the QPC. Since the difference of spin density is anti-symmetric with respect to the middle of the narrow portion of the QPC (*y* = *w*/2), there is no net polarization in the QPC channel^40^. The application of asymmetric bias voltage between the QPC SGs leads to a population of spin-up electrons on the sidewall I, which is larger than the population of spin-down electrons on the sidewall II. This leads to an imbalance in the spin density profiles and results in a net spin-up polarization in the narrow portion of the QPC. The presence of a strong repulsive Coulomb e-e interaction enhances this spin imbalance^40^. In the regime of single-mode transport though the QPC, a 0.5 conductance plateau appears as the spontaneous spin polarization can reach near 100% in this regime^40,51^.

In QPCs with symmetrically biased top split-gates, it has been shown that backscattering events destroy the quantized normal conductance plateaus^62–64^. For instance, Laughton *et al*.^63^ have shown that remote impurity scattering from the doped layers in the heterostructures used to build the QPCs is responsible for an indirect mechanism for electron backscattering. This leads to scattering via quasi-localized sates which can occur for any propagating mode and is more pronounced with higher mode occupancy.

**Figure 5:**
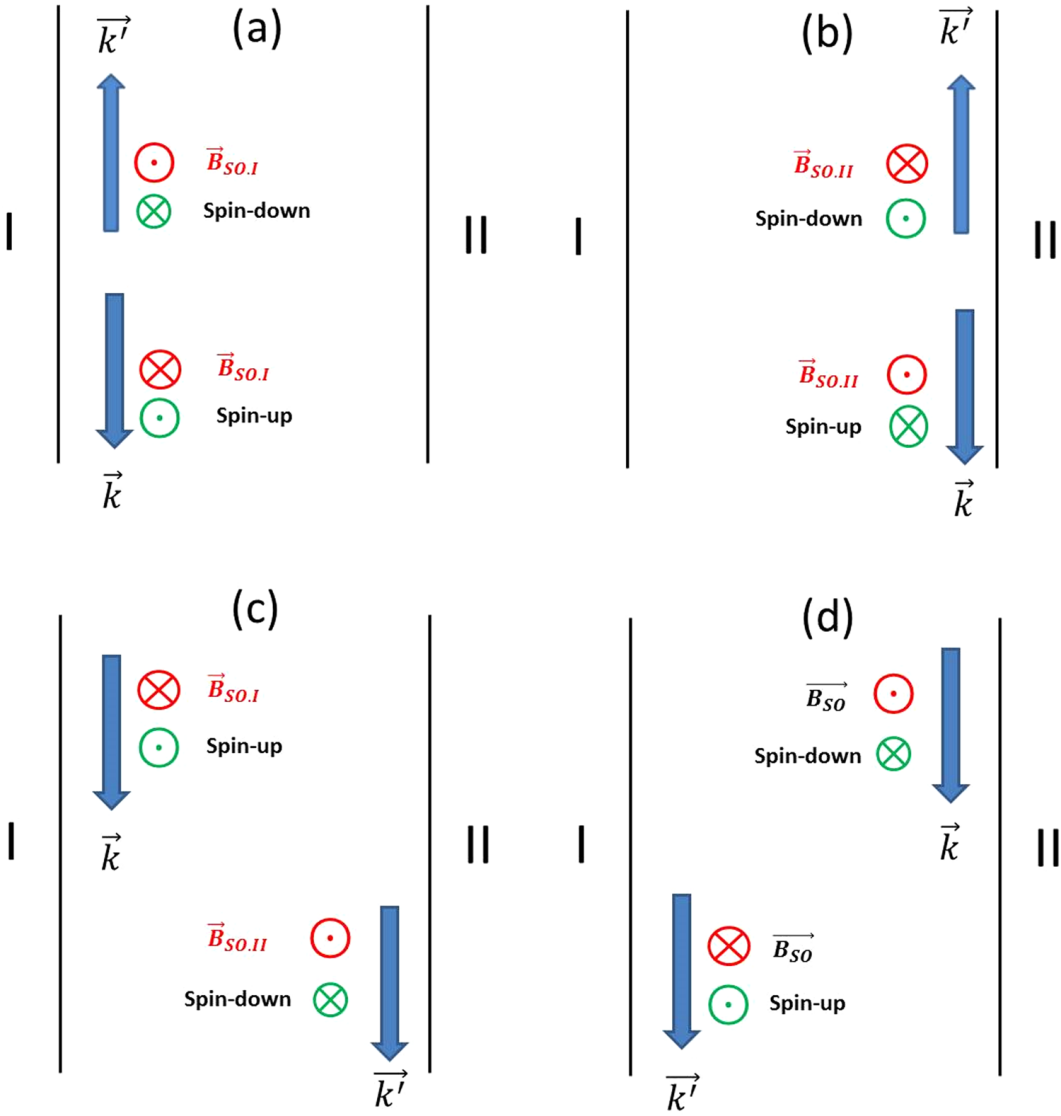
Illustration of various scattering mechanisms leading to a reduction of the amount of spin polarization in the narrow portion of a QPC and accompanying reduction in the hysteresis loops in the conductance plots: back-scattering spin-flip events on (**a**) the left (I) and (**b**) right sidewall (II) of the QPC, (**c**) forward scattering from the left (I) to the right side (II) of the QPC, and (**d**) forward scattering from the right (II) to the left side (I) of the QPC. In the presence of multimode transport, the wave vector of the scattered electron $\overrightarrow{{k}^{\text{'}}}\,$can either be equal or different in magnitude compared to the wave vector $\overrightarrow{k\,}\,$of the electron prior to scattering. In all cases, next to each arrow indicating the direction of the incident and scattered electron, the top and bottom circles illustrate the direction of the effective magnetic field ${\overrightarrow{B}}_{SO}\,$and the direction of alignment of the electron spin along the z axis.

In the presence of LSOC in a narrow channel, non-magnetic impurity scattering can lead to coupling between eigen spinors of spin-up and spin-down electrons (at the same energy) on the same or opposite sides of the QPC channel because these eigenspinors are not orthogonal, a phenomenon similar to what has been reported in the transport properties of spin field effect transistors^65^. As a result, impurity scattering can cause both elastic intra-subband and inter-subband transitions that relax spin and reduce the overall spin polarization in the QPC channel. In our QPCs, we have shown in the past that in addition to remote impurity scattering, surface roughness and impurity (dangling bond) scattering on the sidewalls of the QPC^30^ trigger additional backscattering events. Figure 5 illustrates the different scattering mechanisms leading to a reduction of the spin polarization in the narrow portion of the QPC including: back-scattering spin-flip events on left and right sidewalls of the QPC (Fig. 5a,b), forward scattering from the left (I) to the right side (II) of the QPC (Fig. 5c), and forward scattering from the right (II) to the left side (I) of the QPC (Fig. 5d). In the presence of multimode transport, the wave vector of the scattered electron $\overrightarrow{{k}^{\text{'}}}\,$can either be equal or different in magnitude compared to the wave vector $\overrightarrow{k}$ of the electron prior to scattering. In all panels in Fig. 5, the top and bottom circles next to each arrow showing the direction of the incident and scattered electron indicate the directions of the effective magnetic field ${\overrightarrow{B}}_{SO}$ as well as the directions of alignment of the electron spin along the z axis. The influence of remote impurity scattering increases with the QPC channel width. For a fixed of value of (V_G_ - V_T_),where V_T_ is the specific pinch-off voltage for conduction in a QPC, the electron density in the QPC channel increases with the width of the QPC channel. As a result, there is an increased screening of the electron-electron interaction leading to a less favorable condition for the existence of a spin polarized state in the QPC channel^51^.

## Conclusions

The physical mechanisms responsible for the disappearance of the 0.5G_o_ conductance plateau and the decrease in size of the hysteresis loops in a series of asymmetrically biased InAs based QPCs with varying channel width but identical channel length (as their aspect ratio (width/length) increases) were identified as: (1) multimode transport in the QPC with larger channel leading to spin-flip backscattering events due to both remote impurities in the doping layer of the heterostructure and surface roughness and impurity (dangling bond) scattering on the sidewalls of the QPC constriction, and (2) an increased in carrier density leading to the screening of the e-e interaction in the QPC channel.

In the QPC devices studied here, as their aspect ratio increases, more channels participate in conduction through the narrow portion of the QPC and screening effects are more important in the QPC channel reducing the importance of e-e interaction. As a result, the 0.5G_0_ conductance plateau gradually disappears as the QPC channel width increases, a trend supported by NEGF simulations we reported in the past^51^.

The progressive disappearance of the net spin polarization in the QPC channel and the accompanying reduction in the size of the hysteresis loops as the QPC channel width increases is an another indirect proof of the onset of spin polarization in InAs based QPCs in the presence of LSOC and an asymmetric bias between the QPC SGs.

Asymmetrically biased QPCs with in-plane SGs can act as efficient spin injectors and detectors which could lead to the fabrication of all-electric spin valves. It is anticipated that the asymmetrically biased QPCs studied here could be used as spin-based sensors, filters, and interferometers for future spintronic applications. Furthermore, the onset of hysteresis in the current-voltage characteristics of spin based devices could lead to their potential applications in multilevel logic and data storage circuits^66^.

Towards that goal, it is imperative to explore means to increase the temperature at which QPC spin polarizer can be used for any practical application. InAs has a high intrinsic SOC but a short spin coherence length of around a micron at 4.2 K^61^. The latter is only around tens of nanometers at ambient temperatures. Hence, InAs or any other semiconductor with a large intrinsic SOC, is not suitable for practical applications at room temperature. Our NEGF simulations have shown that even a very weak SOC can cause significant spin polarization provided the electron-electron interaction is very strong^20,51^. Our past finding shows that QPC devices made of GaAs (that possesses a weak intrinsic SOC) can also be useful in generating fully spin polarized current by purely electrical means^28,31^. Some advantages of using GaAs based QPCs include: its long spin coherence length of tens of microns^6^ at ambient temperatures; GaAs samples can be grown with very low electron concentration leading to a strong e-e interaction; The existence of a large Schottky barrier at GaAs/metal interfaces which is beneficial for the deposition of surface gates.

However, one disadvantage of GaAs is that it has a large surface depletion due to the Fermi level pining by surface states^67^. In fact, in our previous experiments with GaAs QPC devices, we had to apply a large positive bias (more than + 10 volts) on both the SGs to first open the QPC constriction at 4.2K. This is a drawback for GaAs based QPC devices if they are to be used in future generations of low power circuits. In contrast, for InAs QPCs, the Fermi level is pinned into the conduction band bottom, making it easier to form Ohmic contacts to the device which facilitates already-open QPC channel at T = 4.2 K and below. There is therefore a need to look into alternative semiconductors and their compounds for asymmetrically biased QPC devices to become operational at temperature much higher than liquid helium.
